# Public knowledge and attitudes toward automated external defibrillators use among first aid eLearning course participants: a survey

**DOI:** 10.1186/s13019-022-01863-1

**Published:** 2022-05-16

**Authors:** Yun-Ming Wang, Li-Ting Lin, Jing-Hao Jiang, Yi Jiang, Xiao-Qing Jin

**Affiliations:** 1 The Emergency Center, Zhongnan Hospital of Wuhan University, Wuhan, 430071 Hubei China; 2 First Clinical College, Wuhan University, Wuhan, 430060 Hubei China; 3 Department of Dermatology and Venereology, Peking University First Hospital, Beijing Key Laboratory of Molecular Diagnosis on Dermatoses, National Clinical Research Center for Skin and Immune Diseases, Beijing, 100034 China

**Keywords:** Automated external defibrillators (AEDs), Out-of-hospital cardiac arrest (OHCA), AED education, Emergencies

## Abstract

**Objective:**

Survival from out-of-hospital cardiac arrest (OHCA) often depends on the effective and immediate use of automated external defibrillators (AEDs). Given that there have been few studies about AED use in China, the purpose of this study is to investigate the knowledge and attitudes regarding AED use among the Chinese public, then provide an effective suggestion for AED education strategies and legislation.

**Method:**

The online survey was conducted among Chinese participants of the First Aid eLearning courses in June 2020.

**Result:**

A total of 2565 (95.00%) surveys were completed, only 23.46% of respondents with non-medical related respondents reported having attended previous AED training courses. Regarding the basic knowledge of AEDs, few respondents (12.28%, n = 315) could answer all four questions correctly. 95.67% (n = 2454) were willing to learn AED use. Even if without the precondition of being skilled in AEDs, the female was more likely to rescue OHCA patients than the male (*p* = 0.003). Almost all respondents (96.65%) showed a strong willingness to rescue OHCA patients with training in using AEDs. The top four barriers to rescuing OHCA patients were lack of practical performing ability (60.47%), fear of hurting patients (59.30%), inadequate knowledge of resuscitation techniques (44.19%), and worry about taking legal responsibility (26.74%).

**Conclusion:**

Our study reflects a deficiency of AED knowledge among the general public in China. However, positive attitudes towards rescuing OHCA patients and learning AED use were observed, which indicates that measures need to be taken to disseminate knowledge and use of AEDs.

**Supplementary Information:**

The online version contains supplementary material available at 10.1186/s13019-022-01863-1.

## Introduction

Sudden cardiac arrest (SCA) is the main cause of sudden death in the world, presenting a huge social burden [1]. Survival rates of individuals who experience SCA remains disappointing, with an estimated 10% for those suffering out-of-hospital cardiac arrest (OHCA) [2]. One of the reasons for low survival of SCA is that it occurs suddenly, with specific pre-arrest conditions unknown [3]. Moreover, in a large majority of cases, emergency medical services (EMS) cannot reach the patient timely [4]. Early effective use of automated external defibrillators (AEDs) and immediate implementation of cardiopulmonary resuscitation (CPR), especially by laypersons, are the best strategies to improve SCA survival rates [5, 6].

Prompt use of AEDs is critical for the patients’ outcomes, especially within the first 3–5 min in which the shock is applied, quadrupling the survival rate from SCA [7]. The AHA 2015 guidelines recommend that AED programs be implemented in public places (such as airports, casinos, and sports facilities) where the possibility of cardiac arrest is relatively high and to educate the public to use them. It also encourages lay rescuer to use AEDs as soon as possible if it is accessible, especially those trained bystanders [8]. Despite these efforts, however, less than 5% of OHCA patients receive AEDS before the arrival of EMS, and only 15% of patients with SCA would be able to receive public-access defibrillation in Japan [9]. The awareness rate of AED by domestic bystanders is not high enough, and the utilization rate is not high. Studies have shown that the reason for the low usage rate is that impede the implementation of AEDs. The main reasons are the lack of knowledge and willingness to use AEDs. [10]. It has been shown that simple training can efficiently improve confidence in using AEDs [11].

Several surveys have been conducted to investigate knowledge about use of AEDs among the Chinese public [12, 13]. However, few studies were done about the factors affecting public knowledge of AEDs, and willingness to learn and operate AEDs. The aims of our study are to assess knowledge and attitudes toward AED use among Chinese public in the First Aid eLearning courses, and to identify the barriers against their use, to provide an effective suggestion for AED education strategies and legislation.

## Materials and methods

### Study design and participants

The study was approved by the ZhongNan Hospital of Wuhan University Medical Ethics Committee. All data were collected under respondents’ permissions. The data for this survey were acquired via questionnaires distributed to the public through the Internet learning platform. We collected responses from those who had taken our first aid eLearning course (http://www.icourses.cn/cuoc/) between June 2020 and July 2020. We posted questionnaires prior to the CPR course, with individuals younger than 14 years old excluded from this investigation.

### Questionnaires design and distribution

The questionnaire was developed by the research team members with the help of reviewing the literature. The final edition of questionnaire was formed after all questions and pre-coded answer options were reviewed and discussed with the Emergency Department specialists in our hospital. The questionnaire consisted of 3 parts, with 13 questions total. The first part was the respondents’ personal characteristics (age, gender, educational level, occupation, previous AED training). The second part was the survey of the basic knowledge of AEDs: full name, function, indication, and method of use. The last part, which was the core, was respondents’ attitudes, covering the questions: whether they would be willing to rescue OHCA patients whether or not it required skill in using AEDs, whether they would be willing to learn how to use AEDs, and why they might be reluctant to rescue OHCA patients.

### Data analysis

To ensure the effectiveness of the questionnaire, any unfinished questionnaire was excluded. We imported all valid data into a database in SPSS Version 22.0 (IBM Institute, Chicago, USA). The respondents were permitted to give multiple answers to the reason why they were reluctant to rescue the patients, resulting in the proportion summed up to be more than 100%. Descriptive statistics were used to summarize the data. Binary and categorical data were calculated for the frequencies. Chi-square tests were used for univariate analyses. All reported p values were two-tailed, and *p* < 0.05 was considered statistically significant.

## Results

A total of 2700 questionnaires were received, and 2565 sets of data were screened. The completion rate was 95.00%, with 135 questionnaires being ruled out because they were not completed.

### Characteristics of the respondents

More than half were female (56.88%, n = 1459), the predominant age group was 18–35 years old (81.29%), 87.78% were undergraduate degree and below (n = 1872), and 73.29% of respondents were not medical people (n = 1880). Additionally, completion of an AED training course was not frequent among the respondents: the total amount of respondents with previous AED training was 941 (36.39%), 67.01% of medical related respondents had AED training experience (n = 459), while in non-medical related respondents, 74.36% (n = 1398) had not attended AED training before (Table 1).

**Table 1:** Characteristics of the respondents according to AED training status

		AEDª trained	Not AED^a^ trained	
	Overall n = 2565	n = 941 (36.69%)	n = 1624 (63.31%)	*P* value
*Gender*				
Male	1106 (43.12%)	427 (45.38%)	679 (41.81%)	
Female	1459 (56.88%)	514 (54.62%)	945 (58.19%)	
*Age*				
< 18	197 (7.68%)	64 (6.80%)	133 (8.19%)	0.001
18–35	2085 (81.29%)	800 (85.02%)	1285 (79.13%)	
35–50	247 (9.63%)	71 (7.55%)	176 (10.84%)	
> 50	36 (1.40%)	6 (0.64%)	30 (1.85%)	
*Education*				
Undergraduate degree and below	2267 (88.38%)	817 (86.82%)	1450 (89.29%)	
Master degree	268 (10.45%)	112 (11.90%)	156 (9.61%)	
Doctoral degree and above	30 (1.17%)	12 (1.28%)	18 (1.11%)	
*Occupation*				
Medical-related	685 (26.71%)	459 (48.78%)	226 (13.92%)	< 0.001
Non-medical-related	1880 (73.29%)	482 (51.22%)	1398 (86.28%)	
*Will you rescue an OHCA*^*b*^* patient?*				
Yes	2311 (90.10%)	854 (90.75%)	1457 (89.72%)	
No	254 (9.90%)	87 (9.25%)	167 (10.28%)	
*Will you attend an AED training course?*				
Yes	2454 (99.0%)	900 (95.64%)	1554 (95.69%)	
No	25 (1.0%)	9 (4.36%)	16 (4.31%)	

ªAED automated external defibrillator; ^b^OHCA out-of-hospital cardiac arrest

### Public awareness of AEDs use

**Fig. 1:**
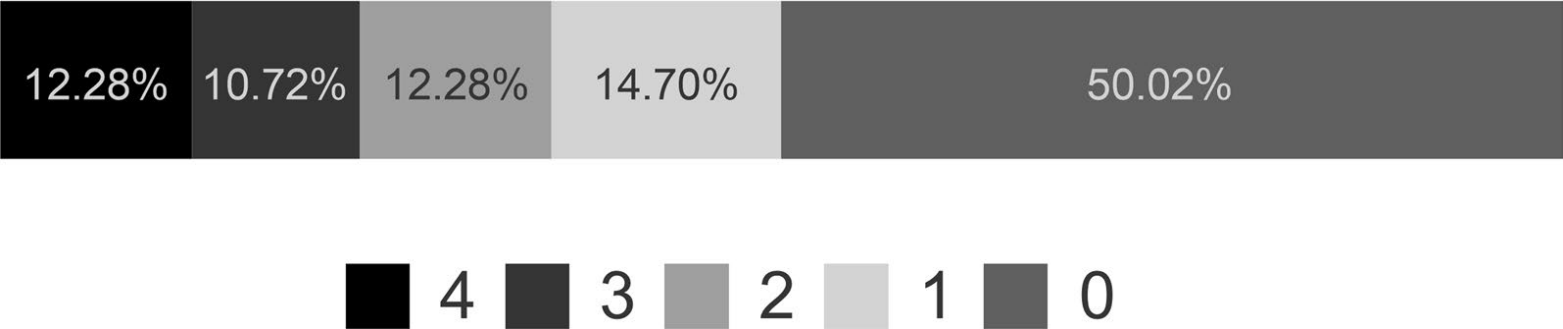
Percentage of correct selections regarding basic AED^a^ knowledge. The numbers represent the number of correct selections chosen by participants, respectively. ^a^AED automated external defibrillator

Of the 2565 respondents, more than 50% had no response to at least one question. Only 12.28% answered all the basic knowledge of AEDs questions correctly (Fig. 1), while even for medical related respondents, only 17.37% had all the correct answers (n = 119), which was higher than non-medical related respondents (10.43%, n = 196, *p* < 0.001).

Respondents with previous AED training showed a significant difference from those with no previous AED training (19.45% and 8.13%, *p* < 0.001). The proportion of correct responses for the questions was fairly poor concerning the full name, function, indication, and method of use of AEDs—respectively, 34.50% (n = 885), 25.81% (n = 662), 37.31% (n = 957), and 22.92% (n = 588). Regarding AED knowledge, medical related respondents and respondents with previous AED training showed a better recognition, but in different gender, age, and educational level, there were statistically significant differences among some of them (Table 2).

**Table 2:** Characteristic of the respondents according to basic AED^a^ knowledge

		Full name of AED		Function of AED		Indication of AED		Use of AED	
		Yes		Yes		Yes		Yes	
	N = 2565	n = 885(%)	*p* value		*p* value		*p* value		*p* value
*Gender*									
Male		364 (32.91%)		300 (29.12%)		396 (35.80%)		266 (24.05%)	
Female		521 (35.71%)		362 (24.81%)		561 (35.45%)		322 (22.07%)	
*Age*									
< 18		64 (32.49%)	0.041	49 (23.35%)		68 (34.52%)		41 (20.81%)	
18–35		702 (33.67%)		531 (25.47%)		783 (37.55%)		488 (23.41%)	
35–50		103 (41.70%)		74 (29.96%)		95 (38.46%)		53 (23.46%)	
> 50		16 (44.44%)		8 (22.22%)		11 (30.56%)		6 (16.67%)	
*Education*									
Undergraduate degree and below		772 (34.05%)		565 (24.92%)	0.016	834 (36.79%)		512 (22.58%)	
Master degree		103 (38.43%)		86 (32.09%)		112 (41.79%)		68 (25.37%)	
Doctoral degree and above		10 (33.33%)		11 (33.33%)		11 (33.33%)		8 (23.67%)	
*Occupation*									
Medical-related		273 (39.85%)	0.001	242 (35.33%)	< 0.001	298 (43.50%)	< 0.001	214 (31.24%)	< 0.001
Non-medical-related		612 (32.55%)		420 (22.34%)		659 (35.05%)		374 (19.89%)	
*Have you attended a previous AED training?*									
Yes		386 (41.02%)	< 0.001	364 (38.68%)	< 0.001	468 (49.73%)	< 0.001	334 (35.49%)	< 0.001
No		499 (30.73%)		298 (18.35%)		489 (30.11%)		254 (15.64%)	

^a^AED automated external defibrillator

### Public attitudes toward learning AED use

95.67% respondents indicated that they would be willing to attend an AED training course. Respondents aged over 50, those having educational level of doctoral degree and above, and those medical related respondents reported a lower willingness to learn AED use, while 96.38% of respondents 35–50 years old expressed the highest willingness to learn AED use. Given the poor percentage of AED training experience in non-medical related respondents, these people showed a higher willingness than the medical related respondents (96.12% and 94.45%, *p* = 0.026). Among respondents with or without previous AED training, there was no statistical difference in the willingness to rescue OHCA patients (*p* = 0.396) and to learn AED use (*p* = 0.944). See Additional file 1: Fig. S1 “Characteristic of the respondents according to basic AED knowledge, attitude and willingness to AEDs” for comprehensive image analysis for the respondents characteristics.

### Public willingness to rescue OHCA patients

**Fig. 2:**
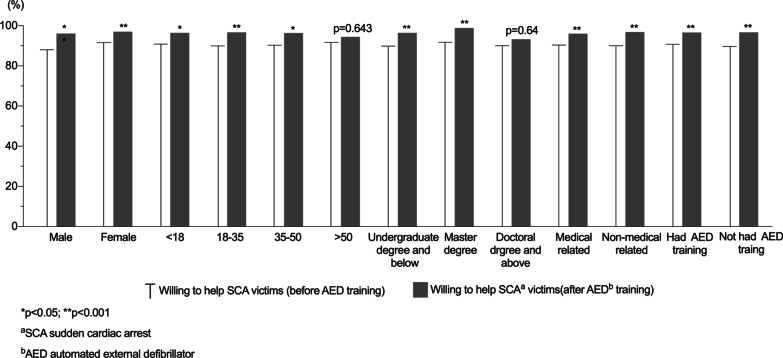
Willingness to help the SCA^a^ patients before/after AED^b^ training. ^a^SCA sudden cardiac arrest; ^b^AED automated external defibrillator

Overall, the majority (90.10%) of the respondents would like to rescue OHCA patients, regardless of whether they had attended previous AED training (AED trained 90.75%, not AED trained 89.72%). Figure 2 showed that the willingness to help SCA victims had been improved in most subgroups after our eLearning course, including male, female, aged under 50, below doctoral degree, medical and non-medical related respondents, with or without previous AED training (*p* < 0.05), There were no statistical significant differences in the willingness of respondents aged over 50 (*p* = 0.643), and had educational level of doctoral degree and above (*p* = 0.64), but it showed a trend of improvement after the training (Fig. 2).

### Reasons for being unwilling to rescue OHCA patients

For the entire survey, though most people (n = 2454) showed great willingness to learn how to use AEDs, a smaller proportion of 90.10% (n = 2311) stated they would be willing actually to rescue OHCA patients. There were also 9.90% of respondents (n = 254) unwilling to try to rescue. The top four reasons given were lack of practical performing ability (60.47%), fear of hurting patients (59.30%), inadequate knowledge of resuscitation techniques (44.19%), and worry about taking the legal responsibility (26.74%). All reasons are shown in Table 3.

**Table 3:** The rank in reason of selection “other” of people who were unwilling to rescue OHCA^a^ patients

Rank	Reason	n	Percentage (%)
1	Lack of practical performing ability	52	60.47
2	Fear of hurting patients	51	59.30
3	Inadequate knowledge of resuscitation techniques	38	44.19
4	Worry about taking the legal responsibility	23	26.74
5	Unwillingness to contact strangers	13	15.12
6	Fear of being swindled	5	5.81
7	No professional license and ability	2	2.33
8	The situation is dangerous to both sides	1	1.16

^a^OHCA out-of-hospital cardiac arrest

## Discussion

The 2015 AHA (American Heart Association) CPR guidelines show that prompt CPR and early defibrillation can effectively improve the survival rate of OHCA patients [8].

In this questionnaire-based survey among the First Aid eLearning courses participats, we found that only 12.28% mastered the AED knowledge and 86.08% (n = 1398) of non-medical related respondents had not previously attended AED training. Most respondents (90.10%) claimed that they would rescue the OHCA, whether they had attended previous AED training before or not (AED trained 90.75%, not AED trained 89.75%). 95.67% of respondents indicated that they would be willing to learn AED use. The top three reasons that they were reluctant to carry out the rescue were lack of practical performing ability (60.47%), fear of hurting patients (59.30%), and inadequate knowledge of resuscitation techniques (44.19%). Additionally, after this online training course, the willingness to perform AEDs has improved among most people.

### Public knowledge of AEDs use

OHCA is a significant health danger world-wide, but the mortality rates of different countries are significantly unequal, which results from discrepancy of AED penetration. Our study revealed a result that 34.50% of respondents knew what AED stands for, compared with 31.71% in Poland [14], and 44.5% in Slovenia [15]. In addition, half (50.02%) of the respondents could answer none of the questions about knowledge of AEDs use while merely one eighth (12.28%) could answer all the questions correctly. Considering these questions are fundamental to AEDs use, the key deficiencies as indicated might partly contribute to the low performance rate of bystander AED and thus low survival rate of OHCA in China, in view of studies showed that adequate knowledge leads to more positive attitudes toward performing CPR and AEDs [16, 17]. In fact, AED training programs have been developed in many countries, such as the United States [18], Sweden and Japan [19, 20], resulting in a great improvement of survival rate of OHCA patients. But we still face some intractable problems, such as low AED penetration, public lack of knowledge, and lack of awareness of first aid skills.

Another finding is that only 17.37% medicine related respondents could answer all questions about AEDs use correctly, slightly higher than those who are not (10.43%), astonishingly low when compared with Japan, where most medical students are familiar with AEDs [21]. In China, CPR training course is compulsory education for all medical students, whereas merely 67.01% of medical related respondents in our survey had attended a previous AED training course, possibly because most respondents had not yet arranged to learn CPR and AED use before our survey, and would study it in future curriculum.

### AED training and age aspects in knowledge of AEDs

The proportion and ways of AED training vary in different countries. We found 36.69% had attended a previous AED training course in our survey, versus 4.9% and 15.2% in 2016 among Chinese public [22, 23], revealing a great improvement on knowledge penetration these years. However, those figures fall short of that published in Japan, Norway, and Slovenia where the proportion of people who have received training in resuscitation techniques more than once were 70%, 89%, and 64% respectively. [13, 21, 24]. Such courses have become a compulsory curriculum [15]. Not surprisingly, respondents with previous AED training proved to have better knowledge of AEDs, but only 19.45% could answer all questions correctly. One possible reason is that their last training course was some time ago. Another reason is the training course is not effective enough to provide proper instruction in operating AEDs. Many studies demonstrated that repeated CPR training can strengthen not only the effectiveness of training courses but also the confidence and willingness to perform bystander resuscitation, and that the optimal interval between classes should be less than 5 years [25, 26]. In a whole, AED training should be implemented as a part of compulsory education, not only with proper instrument, but also with professionals to offer correct information. In addition, the first aid education is not accomplished of an action, multiple training in an appropriate interval is necessary.

After stratification into four age groups (< 18 years; 18–35 years; 35–50 years; > 50 years), our study also showed that respondents of 18–35 years old account for the highest proportion in having previous AED training (38.37%), consistent with other studies, while a huge gap was observed between of respondents willing to learn(95.67%) and respondents actually having been trained(38.37%). This discrepancy can obviously be associated with few approaches of AED knowledge dissemination in Chinese public. According to the major reasons for respondents unwilling to attend a training course were “not knowing where the CPR training courses are” and “a lack of time and concern” [27], it is a possible way to add compulsory education about first aid techniques and refresh these classes to develop both recognition and awareness of importance.

However we found that respondents of 35–50 years old had the best knowledge of AEDs rather than the most trained respondents among 18–35 years old, which is inconsistent with a previous study in Poland [14]. This may be because people 35–50 years old are more likely to suffer heart disease, so they will subconsciously pay more attention to related knowledge. Moreover, another fact is that people use television (39%) and books (29%) rather than first aid training courses as the predominant ways to learn CPR and AEDs in China [28], which may infer the insufficiency of training effectiveness. Therefore, even though the 18–35 years old group had a higher previous training rate, their knowledge of AEDs may not be guaranteed to be better. In many parts of China, the education of first aid techniques are drawn back by the lack of proper instructions and equipment, resulting in much less effective AED classes. Even for those who have attended AED training and had truly mastered the performance, the oblivion after years could be an obstacle when performing first aid to OHCA victims.

### Willingness and influencing factors in helping OHCA

Our results indicated that 90.10% respondents were willing to rescue OHCA patients, regardless of their relationship with the patients and whether it is obliged to perform CPR with mouth-to-mouth ventilation. This percentage is much higher than those in previous studies [29, 30]. Interestingly, although the knowledge of AEDs was significantly different between trained and untrained respondents, we observed no difference of willingness to rescue between these two groups, which is consistent with other studies [21, 23]. Differing from other studies [31–33], we found females were more willing to rescue OHCA patients than males regardless of the required skill (*p* < 0.05). We assume that this difference in gender is owing to their different principal concern, as shown in other studies that more males feared legal dispute while more females lacked confidence [22, 27]. Despite a bit of differences between people who are medical related or not, the Chinese public still express high willingness to help OHCA patients.

### The reasons for reluctance to rescue OHCA patients

More than 11% of our respondents refused to help OHCA victims, and we highlighted various reasons why respondents were reluctant to rescue OHCA patients. The top three reasons were lack of practical performing ability (60.47%), fear of hurting patients (59.30%), and inadequate knowledge of resuscitation techniques (44.19%). We assume all reasons above can be summarized as ‘lack of confidence of precise implementation’. Training in resuscitation techniques has been proven to improve public knowledge and positive attitudes toward bystander CPR and operating AEDs. In this way, the public first aid education is of an urgent need and more professional and comprehensive training programs should be taken into action.

The following reasons (36.04%) included legal responsibility, not professionally licensed, and fear of being swindled. Since the General Principles of the Civil Law of China was put into effect in 2017, there is a law to protect rescuers from liability when their attempts to rescue lead to certain kind of damage to the OHCA patients in the process, which might markedly eliminate these kinds of concerns. Similar regulations, like the “Good Samaritan Law” in the United States, Canada and many European countries, guaranteeing rescuers legal rights, promoting interest and sense of security of bystander CPR and AED operations, have exerted a great effect on improving the survival rate of OHCA patients [27]. With the improvement of the 2017 law, it is predictable that less concern for legal responsibility will be seen in the near future.

Other reasons for reluctance is unwilling to contact with strangers (15.12%). We infer that the fear of disease transmission and feeling embarrassed contribute to the psychological rejection, which have been mentioned in other studies [21, 22, 24]. The proportion of respondents worried about disease transmission was 3% in Japan [21], 16% in Tianjin, China, and 46% in Norway [22, 24]. Some cultural or psycho-social factors have been reported to be relevant to the declining to rescue due to feeling embarrassed in China [22]. Therefore, how to combine resuscitation measures with Chinese traditional customs effectively remains a problem at present.

## Limitations

The main limitation of this study is the selection of the respondents. A high proportion of young people in the respondents were investigated by network questionnaires, which may lead to the results could not fully reflect the average level of present condition. However, this group of people will be the main body of the future population, so the results could reflect the level of the condition in the near future.

## Conclusions

In summary, despite the inadequate knowledge of AEDs, a majority of respondents showed a positive attitude towards rescuing OHCA patients in an emergency situation, and accordingly was willing to learn AED use. AED training can improved the public willingness to perform AED. Our findings will contribute to taking the next step in disseminating knowledge of AED use in the Chinese public.

## Supplementary Information

**Additional file 1**.Public attitudes toward learning AED use.

## Data Availability

The Excel file of questionnaire data used to support the findings of this study are available from the corresponding author upon request.

## Abbreviations

OHCA: Out-of-hospital cardiac arrest
AEDs: Automated external defibrillators
EMS: Emergency medical services
SCA: Sudden cardiac arrest
