# Supplementary figures and images for: Public knowledge and attitudes toward automated external defibrillators use among first aid eLearning course participants: a survey
Source: J Cardiothorac Surg. 2022 May 16;17:119. doi: 10.1186/s13019-022-01863-1 (PMC9112448; doi:10.1186/s13019-022-01863-1)

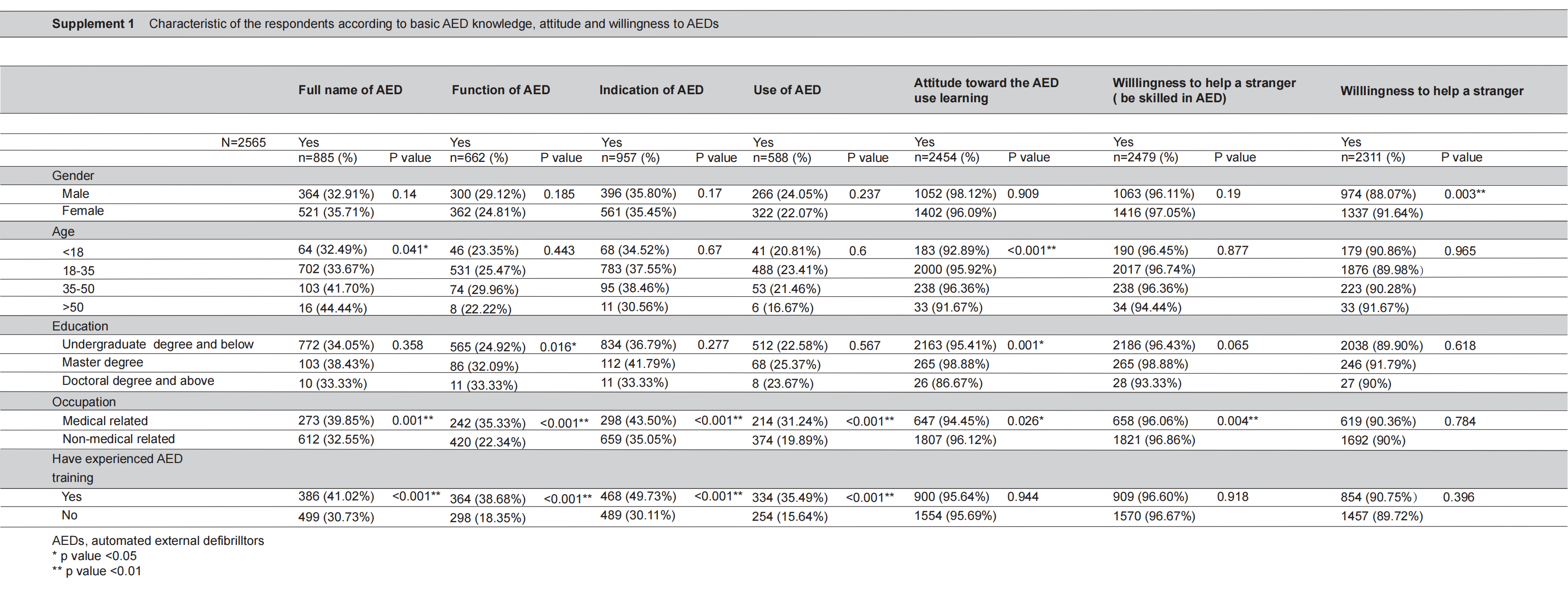

Supplement: Supplementary file 1 — Additional file 1.Public attitudes toward learning AED use. [file 13019_2022_1863_MOESM1_ESM.tif]
